# Alterations in SUMOylation of the hyperpolarization‐activated cyclic nucleotide‐gated ion channel 2 during persistent inflammation

**DOI:** 10.1002/ejp.1606

**Published:** 2020-06-14

**Authors:** Lori A. Forster, Leslie‐Anne R. Jansen, Myurajan Rubaharan, Anne Z. Murphy, Deborah J. Baro

**Affiliations:** ^1^ Department of Biology Georgia State University Atlanta GA USA; ^2^ Neuroscience Institute Georgia State University Atlanta GA USA

## Abstract

**Background:**

Unilateral injection of Complete Freund's Adjuvant (CFA) into the intra‐plantar surface of the rodent hindpaw elicits chronic inflammation and hyperalgesia in the ipsilateral hindlimb. Mechanisms contributing to this hyperalgesia may act over multiple time courses and can include changes in ion channel expression and post‐translational SUMOylation. Hyperpolarization‐activated, cyclic nucleotide‐gated (HCN) channels mediate the hyperpolarization‐activated current, I_h_. An HCN2‐mediated increase in C‐nociceptor I_h_ contributes to mechanical hyperalgesia in the CFA model of inflammatory pain. Changes in HCN2 post‐translational SUMOylation and protein expression have not been systematically documented for a given dorsal root ganglia (DRG) throughout the time course of inflammation.

**Methods:**

This study examined HCN2 protein expression and post‐translational SUMOylation in a rat model of CFA‐induced hindpaw inflammation. L5 DRG cryosections were used in immunohistochemistry experiments and proximity ligation assays to investigate HCN2 expression and SUMOylation, respectively, on days 1 and 3 post‐CFA.

**Results:**

Unilateral CFA injection elicited a significant bilateral increase in HCN2 staining intensity in small diameter DRG neurons on day 1 post‐CFA, and a significant bilateral increase in the number of small neurons expressing HCN2 but not staining intensity on day 3 post‐CFA. HCN2 channels were hyper‐SUMOylated in small diameter neurons of ipsilateral relative to contralateral DRG on days 1 and 3 post‐CFA.

**Conclusions:**

Unilateral CFA injection elicits unilateral mechanical hyperalgesia, a bilateral increase in HCN2 expression and a unilateral increase in post‐translational SUMOylation. This suggests that enhanced HCN2 expression in L5 DRG is not sufficient for mechanical hyperalgesia in the early stages of inflammation and that hyper‐SUMOylation of HCN2 channels may also be necessary.

**Significance:**

Nociceptor HCN2 channels mediate an increase in I_h_ that is necessary for mechanical hyperalgesia in a CFA model of chronic pain, but the mechanisms producing the increase in nociceptor I_h_ have not been resolved. The data presented here suggest that the increase in I_h_ during the early stages of inflammation may be mediated by an increase in HCN2 protein expression and post‐translational SUMOylation.

## 1 INTRODUCTION

Nociceptor signalling is increased in chronic pain states due, in part, to maladjustments in sensory neuron ionic conductances (Berta, Qadri, Tan, & Ji, 2017; Gold & Gebhart, 2010; Pace et al., 2018; Reichling & Levine, 2009). In most cases, the molecular and cellular processes leading to this peripheral sensitization are poorly understood. Mounting evidence suggests that widespread alterations in ion channel SUMOylation may contribute to conductance changes underpinning hyperalgesia associated with chronic pain.

Small ubiquitin like modifier (SUMO) is a ~12 kDa peptide that is reversibly conjugated to lysine (K) residues of target proteins (Flotho & Melchior, 2013). The majority of SUMOylation (~65%) occurs within identifiable consensus sequences (Hendriks, D'Souza, Chang, Mann, & Vertegaal, 2015). The phosphorylation status of a target protein often determines its ability to be SUMOylated (Dustrude et al., 2016). Additionally, the level of target protein SUMOylation depends upon the ratio of 2 opposing enzyme activities: conjugation by ubc9 and deconjugation by isopeptidases, the best studied being the SENP family (isoforms 1–7; Kunz, Piller, & Muller, 2018). A variety of E3 proteins can also stabilize ubc9‐target protein interactions to promote SUMOylation (Flotho & Werner, 2012; Koidl et al., 2016; Werner, Flotho, & Melchior, 2012). Of the four SUMO isoforms, SUMO1–3 are well‐studied but the physiological relevance of SUMO4 is unclear (Watts, 2013). SUMO2 and SUMO3 are 97% identical, and are referred to as SUMO2/3. SUMO1 shares 47% identity with SUMO2/3. Non‐mutually exclusive consequences of target protein SUMOylation include: (1) prevention of other modifications that occur on the same K (Anderson, Eom, & Stover, 2012); (2) binding to phosphoinositides (PIPs) concentrated in the trans‐Golgi [PI(3)P] and plasma membrane [PI(3,4,5)P3] (Arendt et al., 2010; Hammond & Burke, 2020; Kunadt et al., 2015); (3) prevention of protein‐protein interactions through steric hindrance (Dustrude et al., 2016); and most commonly, (4) promotion of protein‐protein interactions through binding domains in partner proteins that recognize SUMO (Psakhye & Jentsch, 2012; Seifert, Schofield, Barton, & Hay, 2015).

Extracellular signalling and neuronal activity regulate the location and activity of the SUMOylation machinery and the SUMOylation status of target proteins (Craig et al., 2012; Hendriks et al., 2015; Loriol, Khayachi, Poupon, Gwizdek, & Martin, 2013; Parker, Forster, & Baro, 2019; Seifert et al., 2015). Both extracellular signals and nociceptor activity are altered during Complete Freund's Adjuvant (CFA)‐induced persistent inflammation, and a generalized increase in SUMOylation is observed in the dorsal root ganglia (DRG; Wang et al., 2018b). SUMOylation of TRPV1 channels is necessary for thermal hyperalgesia during CFA‐induced persistent inflammation, and increasing SUMOylation by genetic knock‐out of SENP1 in sensory neurons exacerbates thermal hyperalgesia during CFA‐induced persistent inflammation (Wang et al., 2018b). SUMOylation of the NaV1.7 auxiliary subunit, CRMP2, prevents channel endocytosis (Dustrude et al., 2016). CRMP2 was hyper‐SUMOylated in primary sensory afferents in a rat spared nerve injury model of chronic neuropathic pain, and blocking CRMP2 hyper‐SUMOylation prevented mechanical and thermal hyperalgesia in this model (Dustrude, Wilson, Ju, Xiao, & Khanna, 2013; Dustrude et al., 2016; Francois‐Moutal et al., 2018; Moutal et al., 2017). These data suggest that nociceptor ion channel hyper‐SUMOylation contributes to hyperalgesia during chronic inflammatory and neuropathic pain.

Several ion channel subunits can be SUMOylated. Existing data generally suggest that hyper‐SUMOylation of ion channel subunits increases cell excitability. Enhancing K^+^ channel SUMOylation reduces outward currents mediated by Kv4 (Welch, Forster, Atlas, & Baro, 2019), Kv11 (Steffensen, Andersen, Mutsaers, Mujezinovic, & Schmitt, 2018), Kv7 (Qi et al., 2014; Xiong et al., 2017), Kv2 (Plant, Dowdell, Dementieva, Marks, & Goldstein, 2011) and K2P1 (Plant et al., 2010; Rajan, Plant, Rabin, Butler, & Goldstein, 2005). To the best of our knowledge, only Kv1.5 does not fit this pattern and is increased by hyper‐SUMOylation (Benson et al., 2007), however, Kv1.5 is not highly expressed in nociceptors (Zheng et al., 2019). Conversely, inward conductances are generally enhanced by hyper‐SUMOylation, including conductances mediated by hyperpolarization‐activated, cyclic nucleotide‐gated 2 (HCN2; Parker et al., 2017), NaV1.2 (Plant, Marks, & Goldstein, 2016), NaV1.7 (Dustrude et al., 2016) and TRPV1 (Wang et al., 2018a). While SUMO modulation of ion channels may be much more complex and vary according to which sites on the ion channel are SUMOylated (Welch et al., 2019), the data suggest a generalized increase in nociceptor ion channel SUMOylation could lead to increased excitability.

The nociceptor hyperpolarization‐activated current (I_h_) is increased in several models of chronic inflammatory and neuropathic pain (Djouhri et al., 2015; Emery, Young, Berrocoso, Chen, & McNaughton, 2011; Schnorr et al., 2014; Weng, Smith, Sathish, & Djouhri, 2012). I_h_ plays a pivotal role in shaping neuronal excitability and synaptic integration by influencing several neuronal activity features including membrane potential, firing threshold, resonance frequency, temporal summation and synaptic strength (Hutcheon & Yarom, 2000; Shah, 2014; Wahl‐Schott & Biel, 2009). Hyperpolarization‐activated cyclic nucleotide‐gated ion channels mediate I_h_ (Sartiani, Mannaioni, Masi, Novella Romanelli, & Cerbai, 2017). There are four HCN isoforms. Genetic ablation of HCN2 in primary sensory afferents prevented mechanical and thermal hyperalgesia in a chronic constriction injury model of neuropathic pain (Emery et al., 2011) and mechanical hyperalgesia in a CFA model of persistent inflammation (Schnorr et al., 2014). HCN2 protein expression increases in small DRG neurons during CFA‐induced persistent inflammation (Acosta et al., 2012; Weng et al., 2012). HCN2 is SUMOylated by both SUMO1 and SUMO2/3 in the rodent brain (Parker et al., 2017). SUMOylation of HCN2 at K669 increases channel surface expression and I_h_ in a heterologous expression system (Parker et al., 2017). Here we investigate changes in HCN2 expression and SUMOylation in rat L5 DRG on days 1 and 3 of CFA‐induced inflammation.

## 2 MATERIALS AND METHODS

### 2.1 Animal ethics

Ethics approval was obtained from the Institutional Animal Care and Use Committee at Georgia State University, and all experiments were performed in compliance with Ethical Issues of the International Association for the Study of Pain and National Institutes of Health (NIH). Male Sprague‐Dawley rats were pair‐housed on a 12‐hr light/dark cycle (lights on at 0700 hr) with ad libitum access to food and water.

### 2.2 CFA model and tissue preparation

Sixty‐day‐old male Sprague‐Dawley rats were injected with 200 µl of CFA (Sigma, F5881, 1 ml contains 1 mg of heat‐killed and dried *Mycobacterium tuberculosis* [strain H37Ra, ATCC 25,177], 0.85 ml paraffin oil and 0.15 ml of mannide monooleate) into the mid‐plantar surface of the right hindpaw. Control rats were handled, but did not receive CFA injections. Either 1 or 3 days later rats were anesthetized with 600 µl of sodium pentobarbital (390 mg/ml) in phenytoin sodium (50 mg/ml) followed by an intracardiac injection of heparin (NDC 25021‐400‐30, 1,000 USP units/ml). Animals were perfused with ~200 ml of sodium nitrite saline followed by 350 ml of 4% paraformaldehyde. Hindpaws were measured. CFA‐treated rats showed a significant increase in ipsilateral hindpaw width relative to contralateral hindpaw width (*p* = 0.0003). Control rats showed no change (*p* > 0.9999). After fixation, bilateral L5 DRG was identified by counting down from T13 (located at the last rib) to L6. L5 DRG were dissected and placed into 18% sucrose at 4ºC overnight. The next day, the epineurium of each DRG was removed. DRG were embedded in 0.3% gelatin and sliced in 20 µm cryosections (Leica CM3050 S); slices were stored at 4ºC for short‐term storage (<2 months), or at −80ºC for longer‐term storage.

### 2.3 Antibodies and reagents

The HCN2 polyclonal rabbit antibody (Alomone, APC‐030) concentration used was 1:200 for proximity ligation assays (PLA), 1:250 for immunohistochemistry (IHC) experiments, 5 μg for immunoprecipitations (IP), and 1:1,000 for Western Blots. A secondary Alexa 488 goat anti‐rabbit was used at 1:400 (Jackson ImmunoResearch) for IHC experiments. For PLA, a monoclonal mouse antibody against SUMO1 (Santa Cruz, Sc‐5308) was used at 1:100, and a monoclonal mouse antibody against SUMO2/3 (Developmental Studies Hybridoma Bank, SUMO‐2 8A2) was used at a concentration of 1:70 and developed by Matinus, M. at John Hopkins School of Medicine, created by the NICHD of the NIH and maintained at The University of Iowa, Department of Biology. All chemicals were obtained through Sigma‐Aldrich unless otherwise stated.

### 2.4 Immunohistochemistry

A cryosection was encircled with a hydrophobic pen (Vector Laboratories), washed once with 0.1 M PBS, twice with HBSS, and incubated with 3.5 μg/ml of Wheat Germ Agglutinin CF® 594 (Biotium) to stain membranes. The cryosection was serially washed twice with HBSS, once with 0.1 M PBS, and permeabilized with 0.1 M PBS with 0.2% Triton X‐100 (PBS‐T). The cryosection was blocked for 30 min with 10% normal goat serum in PBS‐T and incubated with diluted anti‐HCN2 in PBS‐T overnight at room temperature (RT). The cryosection was serially washed three times with 0.1 M PBS and incubated with Alexa 488 goat anti‐rabbit diluted in 0.1 M PBS for 3 hr at RT. The cryosection was washed three times in 0.1 M PBS and incubated with Dapi (300 nM) for 5 min. Serial washes with 0.1 M PBS were repeated three times, and the cryosection was mounted with ProLong Gold antifade reagent (Thermo‐Fisher Scientific). All incubations were performed in a humidity chamber with a minimum volume of 17 μl per cryosection. IHC experiments were repeated on seven experimental animals and three control animals for day 1, and five experimental animals and four control animals for day 3.

### 2.5 IHC analysis

Blind analyses were performed. DRG images were taken at 5× total magnification using an Olympus BX43 fluorescence microscope and cellSens software. Three images were taken per DRG. Images were analysed with Photoshop. Each image was thresholded to remove the intensity of the sheath by successively increasing the tolerance of the magic wand tool. All remaining cells were considered HCN2 positive and were quantified if they showed a definitive nucleus and visible cell perimeter. Cells not meeting these criteria were ignored. All HCN2 positive cells were selected using the magnetic lasso tool and the diameter and gray mean values for each selection were obtained using the measurement feature. The cells were then classified by diameter: small ≤30 μm, medium 31–40 μm, large >40 μm. The gray mean values for all cells in each neuronal size class were averaged and taken to be the mean intensity for the size class. To measure frequency for each size class, the number of HCN2 positive cells in a class was divided by the total number of cells meeting the quantification requirements.

### 2.6 Proximity ligation assay

Proximity ligation assays were performed using Duolink^®^ in situ Red kit (Sigma‐Aldrich) following manufacturer's instructions. Briefly, DRG cryosections were washed once in 0.1 M PBS, twice in HBSS then incubated with 5 µg/ml Wheat Germ Agglutinin‐FITC (Sigma‐Aldrich) to stain membranes. DRG sections were washed twice with HBSS, once with 0.1 M PBS and permeabilized with PBS‐T. Each cryosection was blocked using 17 µl of Duolink^®^ blocking solution for 30 min at 37ºC. Primary antibodies were diluted in Duolink^®^ antibody diluent, 17 µl was applied to each cryosection and incubated overnight at RT. Slides were washed three times for 15 min in 0.1 M PBS, and incubated with Duolink^®^ anti‐mouse minus and Duolink^®^ anti‐rabbit plus secondaries for 1 hr at 37ºC. Slides were washed twice with Duolink^®^ wash buffer A. The ligation reactions were performed at 37ºC for 30 min. Slides were washed twice for 2 min with wash buffer A. The amplification reaction time was extended by 10% to 110 min at 37ºC. Slides were washed twice for 10 min in wash buffer B then once in 0.1× wash buffer B before mounting with Vectashield non‐hardening mounting medium (Vector laboratories, H‐1000). Slides were sealed with clear nail varnish. All incubations were performed in a humidity chamber. All washes with PLA wash buffers A or B were performed in a minimum volume of 70 ml. PLA experiments using SUMO2/3 were repeated on six experimental animals and three control animals at 1 day, and on seven experimental animals and four control animals at day 3. PLA experiments using SUMO1 were repeated on six experimental animals and four control animals at day 1, and six experimental animals and three control animals at day 3.

### 2.7 PLA analysis

Blind analyses were performed. Images were acquired with a Zeiss 700 confocal microscope using a 40× oil immersion objective within 5 days of the PLA experiment. Three sections were analysed per DRG. For each section, a minimum of 3 z‐stacks was acquired. Optical slices were 0.9 µm thick with an interval of 1 µm. Images were analysed using the FIJI version of ImageJ. A maximum projection was created from 5 z‐slices from the centre of a cell. A projection contained a visible nucleus and clear cell boundaries and did not overlap with neighbouring cells or fibres. Cells were outlined, and thresholded using the triangle method. The triangle method assumes that the intensity of the image is skewed in one direction and finds a value where the skew from background intensity is removed, leaving only signal above that value. Watershed analysis was performed to divide any coalesced signals. The analyse particle feature was used to obtain puncta number, mean pixel intensity (MPI) and area for each punctum, as well as the area of the cell and Feret's diameter. Mean pixel intensity of the puncta within one cell was obtained by a program developed by Alex Perez, to calculate the average mean puncta intensity within one cell and allow us to batch process large amounts of data.

### 2.8 Rat DRG membrane preparation and denaturing IP

Approximately 50 DRG were extracted from two rats and placed in autoclaved Dulbecco's phosphate buffer saline (DPBS; 2.7 mM KCl, 1.5 mM KH_2_PO_4_, 136.9 mM NaCl, 8.9 mM Na_2_HPO_4_) on ice. DPBS was removed and the DRG were mechanically homogenized in 250 µl of ice cold homogenization buffer (0.3 M sucrose, 10 µl of 0.1 M PBS pH 7.4, 1 mM EDTA, 1:100 protease inhibitor cocktail [Sigma, cat. P8340]) supplemented with 20 mM NEM to prevent SUMO deconjugation. The homogenate was collected and nuclei and cellular debris were pelleted by centrifugation at 16500 g for 10 min at 4°C. The supernatant was retained and membranes were separated by ultracentrifugation in a tabletop ultracentrifuge at 40,000 *g* for 90 min at 4°C. The supernatant was removed, and membranes were resuspended in 20 µl of denaturing buffer (1% SDS, 50 mM Tris‐HCl pH 7.4, 5 mM DTT, 1:100 protease inhibitor cocktail [Sigma, cat. P8340], 20 mM NEM). The resuspended membrane pellet was shaken for 1 hr at 4°C to aid in the resuspension of membrane proteins. The membrane fraction was then brought to volume of 200 µl with dH_2_O to dilute the SDS prior to IPs. To further denature proteins, the sample was heated at 95°C for 10 min. The boiled sample was cooled on ice before addition of the IP antibody. ~1,000 µg of protein was used for each IP with 5 µg of rabbit anti‐HCN2 or 5 µg of normal IgG for negative controls. IPs were performed using the Classic Magnetic IP/Co‐IP Kit (Pierce, cat. 88804) according to the manufacturer's instructions. IP products were eluted in a volume of 50 µl.

### 2.9 Western blot

An HCN2 IP and a negative IgG control from the same membrane preparation (see above) were run side by side in sets of 3 on the same 12% SDS‐polyacrylamide gel. After electrophoresis, proteins were transferred from the gel for 2 hr at 45 AMP to a PVDF membrane (Immobilon‐P, cat. #IPVH00010) using a semi‐dry electroblotting system (OWL). The membrane was cut into three pieces and each piece comprised one set of HCN2 and IgG IP products. Membranes were blocked in 5% non‐fat dry milk in TBS (50 mM Tris‐HCl pH 7.4, 150 mM NaCl) for 1 hr at RT. Blots were washed 1× for 10 min with TTBS (TBS + 0.1% Tween20), and then primary antibodies, prepared in 1% non‐fat dry milk in TTBS, were added and incubated overnight at 4°C. Each set was probed with a different antibody that recognized HCN2, SUMO1 (Cell Signaling, 4930S, 1:1,500) or SUMO2/3 (Abcam, ab3742, 1:750). Blots were washed 3× for 5 min in TTBS and then incubated with goat anti‐rabbit alkaline phosphatase conjugated secondary antibody (Jackson ImmunoResearch, 1:4,000), prepared in 1% non‐fat dry milk in TTBS, for 2 hr with agitation at RT. Blots were washed 3× for 10 min with TTBS. The membranes were incubated with alkaline phosphatase substrate (Biorad) for 5 min at RT, and then the membranes were exposed to X‐ray film (MedSupply partners) and the chemiluminescent signals were visualized with a Kodak X‐Omat 2000A imager.

### 2.10 Statistics

All statistical analyses were performed using GraphPad Prism. Data were tested for normality. Normal IHC data were analysed with a one‐way ANOVA followed by a Tukey's post‐hoc test. Normal PLA data were analysed with a paired *t*‐test. Non‐parametric data were analysed with Kruskal–Wallis for IHC or Wilcoxon matched‐pairs signed rank tests for PLA. All values are presented as the mean ± *SEM*. *p* < 0.05 was considered statistically significant.

## 3 RESULTS

### 3.1 Inflammation increases the level of HCN2 expression and the number of cells expressing HCN2

**Figure 1:**
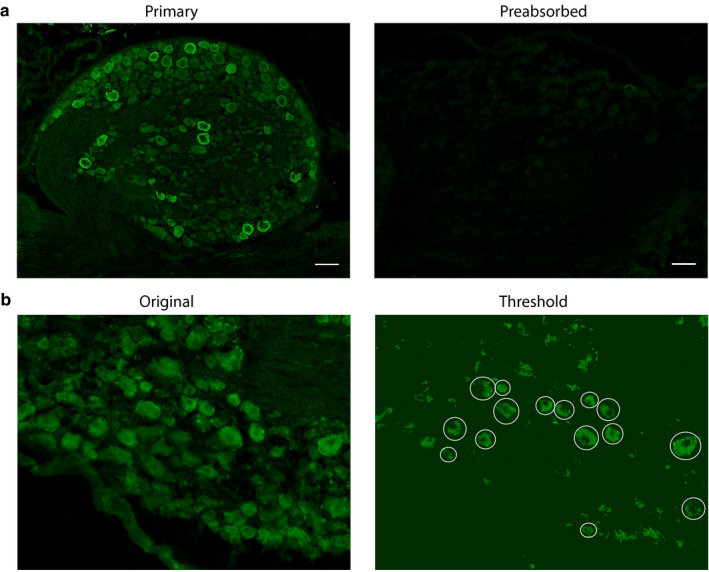
Validation of anti‐HCN2 and quantification of HCN2‐positive cells. (a) Representative IHC. Experiments using antibody and pre‐absorbed antibody show anti‐HCN2 specificity. Scale bars are 100 μm. (b) Representative analysis. Sections show cells before and after thresholding. Cells having an obvious nucleus and plasma membrane, and therefore meeting the criteria for analysis, are circled. HCN2, Hyperpolarization‐activated, cyclic nucleotide‐gated 2; IHC, immunohistochemistry

Previous studies examined the effects of cutaneous hindlimb inflammation in L5 DRG on day 7 post‐CFA and in L4 DRG on days 1 and 4 post‐CFA (Acosta et al., 2012; Weng et al., 2012). This study examined the results of CFA‐induced inflammation in L5 DRG on days 1 and 3 post‐CFA. In the experimental treatment group, CFA was injected into the right hindpaw of each adult male Sprague‐Dawley rat. In the control treatment group, rats were handled but not injected. Animals were perfused on day 1 or 3 post‐CFA injection/handling. L5 DRG were dissected, cryosectioned (20 µm) and used in IHC experiments. The specificity of the HCN2 antibody (anti‐HCN2, Alomone Labs) was previously verified in a rigorous series of experiments (Acosta et al., 2012) and was confirmed here in a series of preliminary IHC experiments using antibody that was pre‐absorbed with the immunogen provided by the manufacturer. Pre‐absorption greatly diminished staining intensity (Figure 1a). As described in Section 2, raw images of IHC experiments (Figure 1b, left panel) were processed, and HCN2‐positive neurons were identified and circled (Figure 1b, right panel). Cells were classified into three groups according to diameter: small (≤30 µm), medium (30 µm < *x *< 40 µm) and large (>40 µm). HCN2 immunoreactivity (IR) was measured as the MPI for each cell and as the frequency of HCN2 expressing cells. The number of cells expressing HCN2 was quantified for each of the three cell sizes by dividing the number of HCN2 positive cells in each size category by the total number of cells in the DRG section (HCN2‐positive and ‐negative) and multiplying by 100. This was termed frequency.

**Figure 2:**
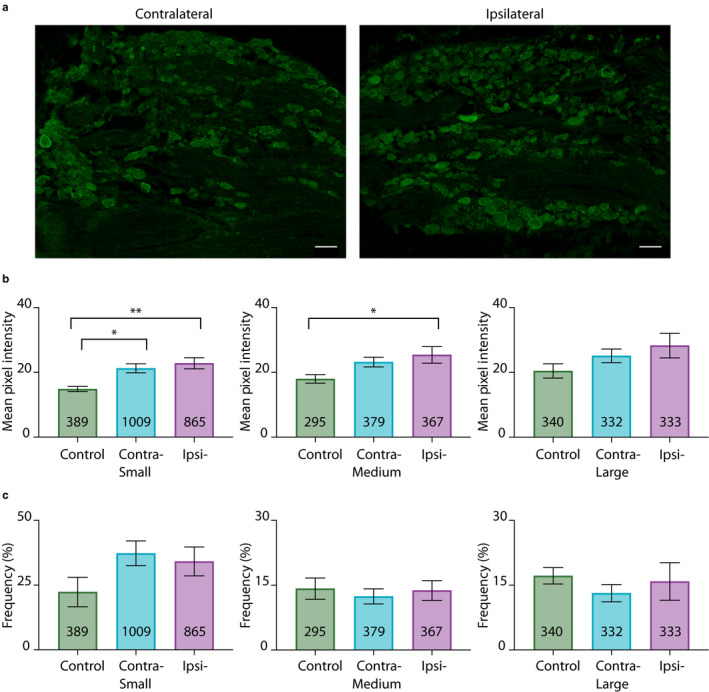
CFA‐induced inflammation alters HCN2 protein expression on 1 day post‐CFA. (a) Representative images of HCN2 IR in contralateral and ipsilateral L5 sections 1 day post‐CFA injection. Scale bars are 100 μm. (b) HCN2 expression is elevated in experimental compared to control DRG. Average mean pixel intensities ± *SEM*s are plotted for three size classes of DRG neurons. A one way ANOVA with a Tukey's post‐hoc test that makes all possible comparisons shows HCN2 IR is significantly elevated in small cells for experimental versus control treatment groups (*F*(2,17) = 8.468; *p* = 0.0028). Asterisks indicate which two‐way comparisons were significant. A one‐way ANOVA with a Tukey's post‐hoc shows HCN2 IR is significantly elevated in medium cells for ipsilateral versus control treatment groups (*F*(2,17)=3.748; *p* = 0.0448). Asterisks indicate which pairwise comparisons were significant: *p < 0.05; **p < 0.01. A one‐way ANOVA shows that HCN2 IR was not significantly different between large cells from experimental and control DRG (*F*(2,17) = 1.794; *p* = 0.1963). (c) HCN2 frequency is unaltered 1 day post‐CFA injection. Bar plots of the frequency of HCN2 positive neurons in each size category ([HCN2‐expressing cells per category ÷ total number of cells in all categories] × 100). Statistical tests show there were no significant differences between experimental and control animals for any cell type (small; KW(2,17) = 3.603; *p* = 0.1682; medium; *F*(2,17) = 0.1860; *p* = 0.8317; large; KW(2,17) = 1.999; *p* = 0.3824). CFA, Complete Freund's Adjuvant; DRG, dorsal root ganglia; HCN2, hyperpolarization‐activated, cyclic nucleotide‐gated 2; IR, immunoreactivity; KW, Kruskal–Wallis

The mean pixel intensities measured on day 1 post‐CFA were compared between control and experimental DRG (Figure 2b). The average MPI for small diameter neurons in experimental animals significantly increased by ~50% in both contralateral (21.3 ± 1.4) and ipsilateral (22.8 ± 1.7) relative to control DRG (14.9 ± 0.8). The average MPI for medium diameter neurons increased by ~40% in experimental (ipsilateral, 25.4 ± 2.6; contralateral, 23.2 ± 1.5) relative to control DRG (18.0 ± 1.3). The increase was statistically significant only for the ipsilateral DRG (Figure 2 legend). There were no significant differences in the average MPI between large diameter neurons (contralateral, 25.1 ± 2.1; ipsilateral, 28.3 ± 3.8; control, 20.5 ± 2.2). A comparison of the mean frequency showed that there were no significant differences between control and experimental animals or between ipsilateral and contralateral DRG for any neuronal size class (Figure 2c). In general, ~55%–65% of all DRG neurons examined here expressed HCN2, and small‐diameter neurons had the greatest number of HCN2‐positive cells (contralateral: small, 37.3 ± 4.8%; medium, 12.4 ± 1.8% large, 13.1 ± 2.0%; ipsilateral: small, 34.1 ± 5.6%; medium, 13.8 ± 2.3%; large, 15.9 ± 4.4%; control: small, 22.3 ± 5.7%; medium, 14.2 ± 2.5%; large, 17.2 ± 1.9%).

**Figure 3:**
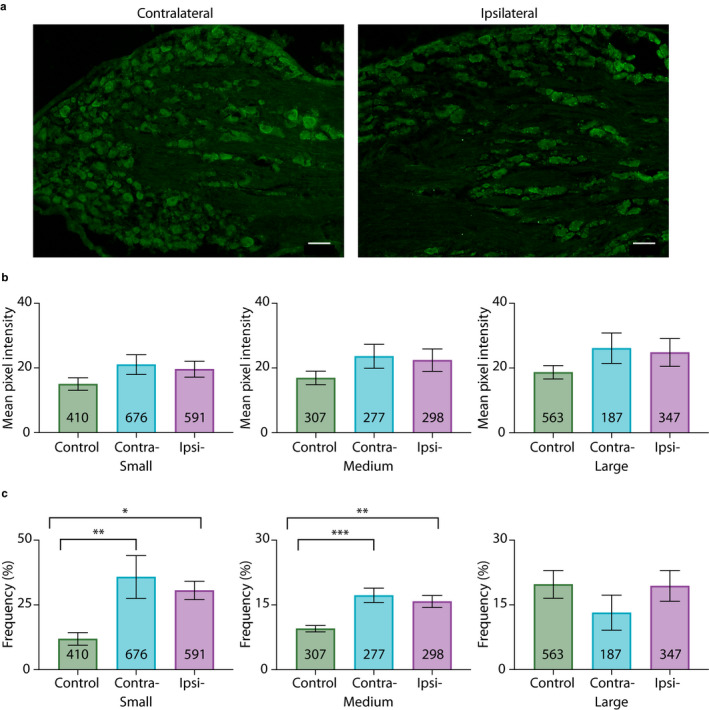
HCN2 protein expression is altered on day 3 post‐CFA. (a) Representative images of HCN2 IR in contralateral and ipsilateral L5 sections 3 days post‐CFA. Scale bars are 100 μm. (b) The intensity of HCN2 IR is unaltered 3 days post‐CFA. Bar plots of mean pixel intensity. One‐way ANOVAs show no significant differences between treatment groups for any size category (small; *F*(2,15) = 1.893; *p* = 0.1849; medium; *F*(2,15) = 1.667; *p* = 0.2220; large; *F*(2,15) = 1.499; *p* = 0.2550). (c) HCN2 frequency is significantly increased in small and medium diameter neurons in experimental compared to control animals. Bar plots of the frequency of HCN2 IR in each size class ([HCN2‐expressing cells per category ÷ total number of cells in all categories] × 100). A one‐way ANOVA with a Tukey's post hoc test shows HCN2 frequency is significantly elevated in small cells for experimental versus control treatment groups (*F*(2,15) = 8.022; *p* = 0.0043). Asterisks indicate which two‐way comparisons were significant. A one‐way ANOVA with a Tukey's post hoc test shows HCN2 frequency is significantly elevated in medium cells for experimental versus control treatment groups (*F*(2,15) = 13.51; *p* = 0.0004). Asterisks indicate significant differences in pairwise comparisons: *p < 0.05; **p < 0.01; ***p < 0.001. A Kruskal–Wallis (KW) test shows HCN2 frequency is not significantly different between large cells for experimental versus control treatment groups (KW(2,15) = 3.058; *p* = 0.2251). CFA, Complete Freund's Adjuvant; HCN2, hyperpolarization‐activated, cyclic nucleotide‐gated 2; IR, immunoreactivity

The situation changed by day 3 post‐CFA such that there was a significant increase in the number but not intensity of cells expressing HCN2 (Figure 3). The average mean pixel intensities were no longer significantly different for any size class (Figure 3b), although there was still a slight elevation in contralateral (small, 21 ± 3.1; medium, 23.6 ± 3.7; large, 26.1 ± 4.7) and ipsilateral DRG (small, 19.6 ± 2.5; medium, 22.4 ± 3.5; large, 24.8 ± 4.3) relative to control DRG (small, 15 ± 1.9; medium, 16.9 ± 2.1; large, 18.7 ± 2.1). On the other hand, the number of cells expressing HCN2 significantly increased in small and medium but not large diameter neurons in experimental relative to control animals (Figure 3c). Mean frequency in both contralateral (small, 35.8 ± 8.3%; medium, 17.2 ± 1.7%) and ipsilateral DRG (small, 30.6 ± 3.5%; medium, 15.8 ± 1.4%) were significantly increased relative to control DRG (small, 11.9 ± 2.4%; medium, 9.5 ± 0.7%), but were not significantly different from one another.

In sum, unilateral hindpaw inflammation initially produced a significant bilateral increase in HCN2 protein expression in small diameter neurons. CFA‐induced inflammation also produced a significant ipsilateral increase in medium diameter neurons. As inflammation progressed, expression levels returned back toward normal, but the number of small and medium diameter cells expressing HCN2 increased bilaterally.

### 3.2 HCN2 is SUMOylated in the rat DRG

**Figure 4:**
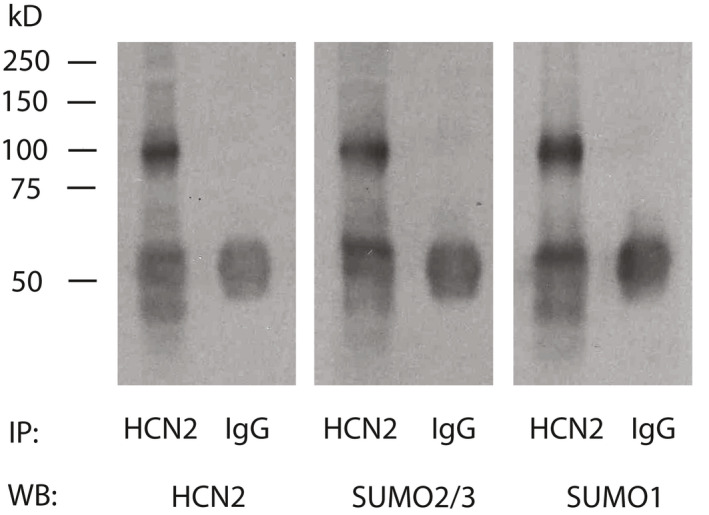
HCN2 is SUMOylated in rat DRG. Denaturing immunoprecipitation (IP) experiments were performed on a DRG membrane preparation with an antibody against HCN2 or IgG (control). IP products from 1 membrane preparation were run in triplicate on an SDS‐polyacrylamide gel followed by western blotting (WB). The blot was cut into 3, and probed for HCN2, SUMO2/3 and SUMO1. The experiment was repeated using three different DRG membrane preparations. A representative result from one experiment is shown. All three WB antibodies recognized the same ~100 kD protein in the HCN2 IP product but not the IgG IP product. The data indicate that SUMO1 and SUMO2/3 are covalently linked to HCN2 since they remained bound under denaturing conditions. The ~50 kD band present in all lanes represents the IP antibody. DRG, dorsal root ganglia; HCN2, hyperpolarization‐activated, cyclic nucleotide‐gated 2; SUMO, small ubiquitin like modifier

SUMOylation of HCN2 channels has never been examined in DRG neurons. To determine if HCN2 channels were SUMOylated in DRG neurons, denaturing IP experiments were performed using DRG membrane preparations and an antibody against HCN2 followed by western blot experiments with antibodies against HCN2, SUMO2/3 and SUMO1. The experiment was repeated three times (i.e., six rats, see Section 2). A representative experiment shows that all three antibodies recognized the same appropriately sized ~100 kD band (Figure 4). This band was not observed in IP experiments using a non‐specific rabbit IgG on the same membrane preparation. In order to prevent non‐covalent interactions between proteins, that is, co‐IP, the membrane preparations were denatured with a high concentration of SDS (1%) and high heat treatment (95°C for 10 min) prior to the IPs (see Section 2). These data indicate that SUMO1 and SUMO2/3 are covalently linked to HCN2 α‐subunits in the rat DRG.

**Figure 5:**
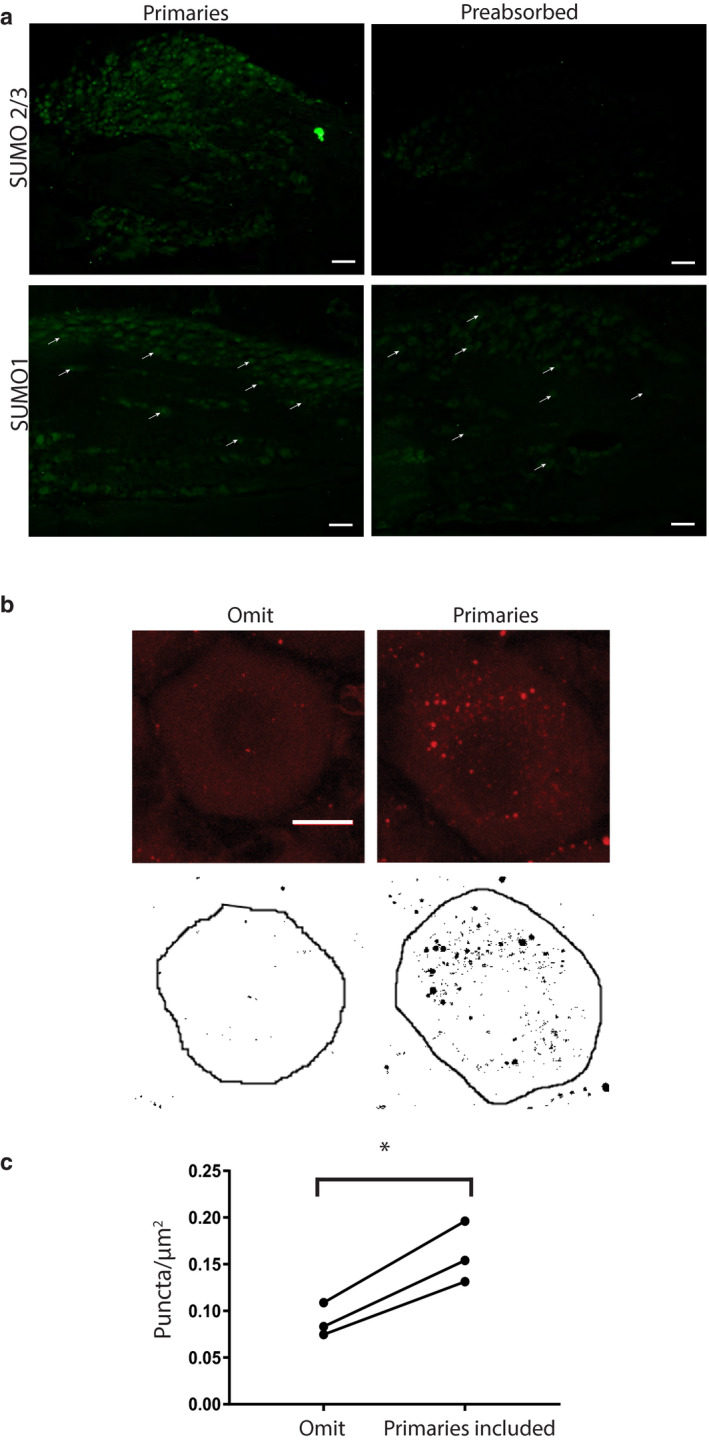
Measuring HCN2 channel SUMOylation. (a) Verification of SUMO antibodies. Antibodies were (right) or were not (left) preabsorbed with the corresponding peptide for SUMO2/3 (upper panel) or SUMO1 (lower panel). SUMO is predominately expressed in nuclei. Note the loss of nuclear staining following preabsorption, for example, white arrows in bottom panel. Scale bars are 100 µm. (b) Method for quantifying HCN2 channel SUMOylation. In situ PLA was performed on DRG cryosections with (experimental) or without (control) antibodies against SUMO2/3 and HCN2. Upper panel shows a 5 µm projection of confocal optical slices through representative cells from control and experimental treatment groups. Each image represents a projection of five slices in continuous succession that together encompass the centre of the cell. Each optical section is 0.9 µm with a z‐stack interval of 1 µm. Note that the red puncta indicating SUMOylated HCN2 channels were largely absent when antibodies were omitted. Scale bar is 10 µm. The cells were circle and thresholded, and the resulting image is shown in the lower panel. Black puncta within the circled region were counted using the analyse particle tool on imageJ and normalized by the area (µm^2^) of the circle. Note that all black puncta, regardless of size were counted. (c) HCN2 channels are SUMOylated in DRG neurons. Plots of puncta per µm^2^ show there are significantly more puncta when primary antibodies for HCN2 and SUMO2/3 were included (0.08 ± 0.01 vs. 0.16 ± 0.02, *p* = 0.0149, paired *t*‐test, *n* = 3, 70 and 62 cells analysed in total) *, p < 0.05. The lines indicate that the cells were from the same experiment, that is, alternate sections from a single DRG on the same slide receiving the same PLA reagents and treated in an identical fashion. DRG, dorsal root ganglia; HCN2, hyperpolarization‐activated, cyclic nucleotide‐gated 2; PLA, proximity ligation assays; SUMO, small ubiquitin like modifier

Next, in situ PLA were used to examine HCN2 channel SUMOylation in individual DRG neurons. Preliminary pre‐absorption experiments on cryosectioned DRG confirmed the specificities of the SUMO1 (anti‐SUMO1) and SUMO2/3 (anti‐SUMO2/3) antibodies (Figure 5a). HCN2 channel SUMOylation was examined using anti‐HCN2 and anti‐SUMO2/3 in PLA on cryosectioned DRG (experimental). Primary antibodies were omitted in control PLA. SUMOylated HCN2 channels appeared as fluorescent puncta in confocal optical sections (Figure 5b, upper panels). Punctum area and intensity varied, which may reflect differences in the number of HCN2 channels represented by a punctum, for example, solitary versus clustered channels, and/or the extent of their SUMOylation. Puncta number and punctum MPI were used as measures of the number of SUMOylated channels and the extent of channel SUMOylation, respectively. To measure HCN2 channel SUMOylation in a single cell, a confocal projection representing 5, ~1 µm optical sections from the centre of a cell was obtained. Note that this represents ~1/4 (small) to 1/8 (large) of the volume of a neuron and that surface sections will be excluded, that is, all plasma membrane staining lies along the perimeter of the cell. A cell was outlined and automatically thresholded to eliminate background (Figure 5b, bottom panels). ImageJ was then used to measure the area of the cell as well as the number and intensity of puncta from within the circled region. Control and experimental PLA were performed on alternating sections of L5 DRG. DRG from three animals were analysed. Results showed significantly more puncta/µm^2^ in experimental versus control treatment groups (Figure 5b). These data indicated that HCN2 channels were decorated with SUMO2/3 in rat L5 DRG and that this method could be used to examine HCN2 channel SUMOylation in individual neurons.

### 3.3 Significantly more HCN2 SUMO2/3 conjugation is observed in small diameter neurons from ipsilateral DRG on day 1 but not day 3 post‐CFA

**Figure 6:**
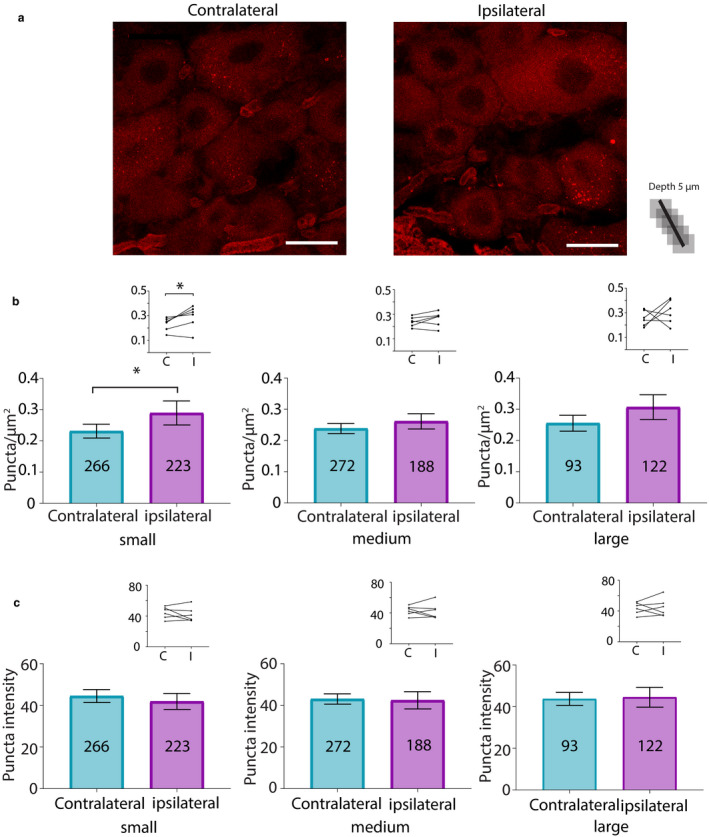
SUMO2/3 conjugation to HCN2 channels is increased in small diameter neurons from ipsilateral relative to contralateral DRG at 1 day post‐CFA. (a) Representative PLA. Scale bars are 25 µm. Note that this image provides an overview, but for the purpose of quantification, individual projections were made for each cell, for example, Figure 5b. (b) Plots of mean number of puncta/µm^2^ for all DRG from six CFA treated animals on day 1 post‐CFA. The number inside the bar represents the total number of cells analysed. Inset: Each line compares the means for contralateral and ipsilateral DRG from one animal. Paired *t*‐tests indicate the number of puncta significantly increased for small but not medium or large neurons in ipsilateral compared to contralateral DRG (small, 0.23 ± 0.02 vs. 0.29 ± 0.04, *p* = 0.0499; medium, 0.24 ± 0.02 vs. 0.26 ± 0.02, *p* = 0.2120; large, 0.26 ± 0.03 vs. 0.31 ± 0.04, *p* = 0.4259), ^*^ *p* < 0.05. (c) Plots of mean puncta intensity on day 1 post‐CFA. Paired *t*‐tests indicate mean puncta intensities were not significantly different between ipsilateral and contralateral DRG for any size category (small, 44.47 ± 3.1 vs. 41.86 ± 3.9, *p* = 0.4495; medium, 43.06 ± 2.5 vs. 42.43 ± 4.1, *p* = 0.8503; large, 43.74 ± 3.1 vs. 44.51 ± 4.8, *p* = 0.8518). CFA, Complete Freund's Adjuvant; DRG, dorsal root ganglia; HCN2, hyperpolarization‐activated, cyclic nucleotide‐gated 2; PLA, proximity ligation assays; SUMO, small ubiquitin like modifier

To test if HCN2 channels were hyper‐SUMOylated during CFA‐induced inflammation, CFA was injected into the right hindpaw of adult male rats. Animals were perfused 1 or 3 days post‐CFA. Bilateral L5 DRG were dissected and cryosectioned. In situ PLA was performed on the cryosections using anti‐HCN2 and anti‐SUMO2/3. Control animals were handled but not injected. HCN2 channels were decorated with SUMO2/3 in small, medium and large neurons from ipsilateral and contralateral DRG 1 day post‐CFA (Figure 6a). The number of puncta per µm^2^ was determined for the three size classes of neurons. Comparisons of control and experimental animals showed no significant differences and are not reported here. On the other hand, paired comparisons between contralateral and ipsilateral DRG from the same experimental animal revealed significant differences. Most likely, differences were revealed in the latter analyses because biological variability was reduced by intra‐animal comparison.

A significant ~25% increase in puncta number was observed in small neurons from ipsilateral relative to contralateral DRG (Figure 6b). No significant differences were observed in medium or large diameter neurons in contralateral versus ipsilateral DRG (Figure 6b). Paired *t*‐tests on left versus right DRG in the control treatment group indicated no significant differences (small, 0.24 ± 0.04 vs. 0.23 ± 0.03, *p* = 0.5389; medium, 0.23 ± 0.04 vs. 0.21 ± 0.06, *p* = 0.6006; large, 0.26 ± 0.03 vs. 0.23 ± 0.04, *p* = 0.6340; *n* = 3 animals). Punctum intensity was divided by punctum area to obtain the MPI per punctum, here termed puncta intensity. The average puncta intensity was calculated for each cell. Average puncta intensity was not significantly different between contralateral versus ipsilateral DRG neurons for any size class (Figure 6c). Control animals showed no differences between left versus right DRG (small, 47.82 ± 2.74 vs. 41.80 ± 4.32, *p* = 0.25; medium, 47.72 ± 2.79 vs. 42.68 ± 4.77, *p* = 0.1694; large, 48.96 ± 2.19 vs. 42.98 ± 5.32, *p* = 0.2730; *n* = 3).

**Figure 7:**
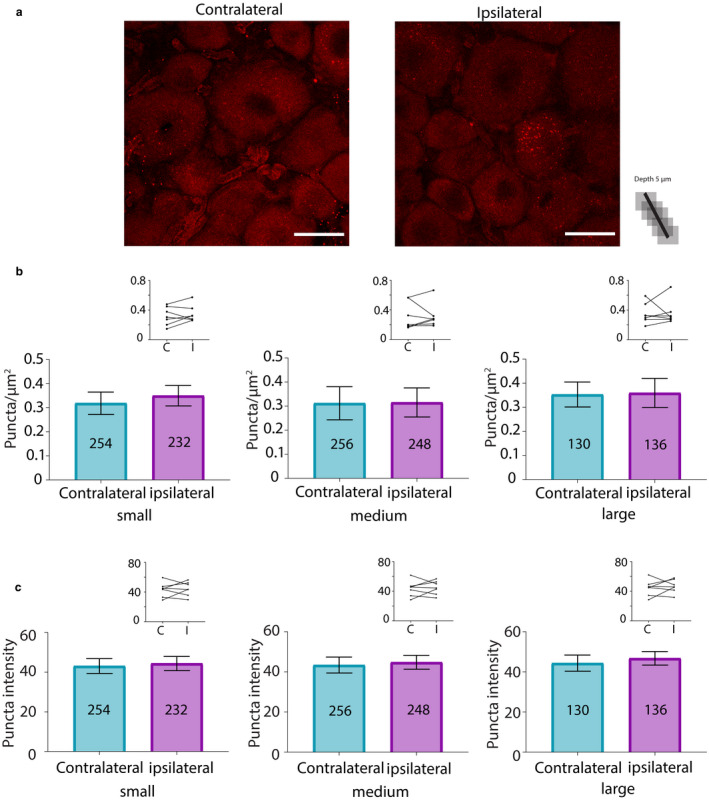
SUMOylation of HCN2 channels by SUMO2/3 does not change 3 days post‐CFA. (a) Representative PLA. Scale bars are 25 µm. Note that this image provides an overview, but for the purpose of quantification, individual projections were made for each cell, for example, Figure 5b. (b) Plots of mean number of puncta/µm^2^ for all DRG from seven CFA treated animals on day 3 post‐CFA. The number inside the bar represents the total number of cells analysed. Inset: Each line compares means for contralateral and ipsilateral DRG from one animal. Wilcoxon matched‐pair tests indicate no significant difference in puncta number for small, medium or large diameter cells (small, 0.32 ± 0.05 vs. 0.35 ± 0.04, *p* = 0.375; medium, 0.31 ± 0.07 vs. 0.32 ± 0.06, *p* = 0.5781; large, 0.35 ± 0.05 vs. 0.36 ± 0.06, *p* = 0.8125). (c) Plots of mean puncta intensity on day 3 post‐CFA. Paired *t*‐tests indicate mean puncta intensities were not significantly different for any size category in ipsilateral compared to contralateral DRG (small, 43.13 ± 3.73 vs. 44.4 ± 3.56, *p* = 0.731; medium, 43.4 ± 3.98 vs. 44.78 ± 3.45, *p *= 0.7087; large; 44.35 ± 4.04 vs. 46.75 ± 3.36, *p *= 0.5670). CFA, Complete Freund's Adjuvant; DRG, dorsal root ganglia; HCN2, hyperpolarization‐activated, cyclic nucleotide‐gated 2; PLA, proximity ligation assays; SUMO, small ubiquitin like modifier

Analyses were performed for animals perfused at 3 days post‐CFA (Figure 7a). There were no significant differences in HCN2‐SUMO2/3 puncta number (Figure 7b) or intensity (Figure 7c) between contralateral versus ipsilateral DRG neurons from experimental animals for any neuronal size class. There were also no significant differences between left versus right DRG in the control treatment group for either puncta number (small, 0.38 ± 0.08 vs. 0.39 ± 0.11, *p* = 0.8946; medium, 0.43 ± 0.11 vs. 0.36 ± 0.12, *p* = 0.2152; large, 0.50 ± 0.10 vs. 0.41 ± 0.11, *p* = 0.0708; *n* = 4) or puncta intensity (small, 71.23 ± 17.97 vs. 61.01 ± 12.95, *p* = 0.1466; medium, 69.65 ± 16.31 vs. 61.14 ± 12.65, *p* = 0.1181; large, 69.74 ± 17.88 vs. 61.03 ± 12.82, *p* = 0.1987; *n* = 4).

In sum, the number of HCN2 channels decorated with SUMO2/3 was transiently increased by ~25% in small neurons from ipsilateral relative to contralateral DRG on day 1 but not 3 post‐CFA. There were no significant changes in the extent of channel SUMOylation.

### 3.4 Conjugation of SUMO1 to HCN2 channels was reduced in medium and large diameter neurons on 1 day post‐CFA, and increased in small neurons on day 3 post‐CFA

**Figure 8:**
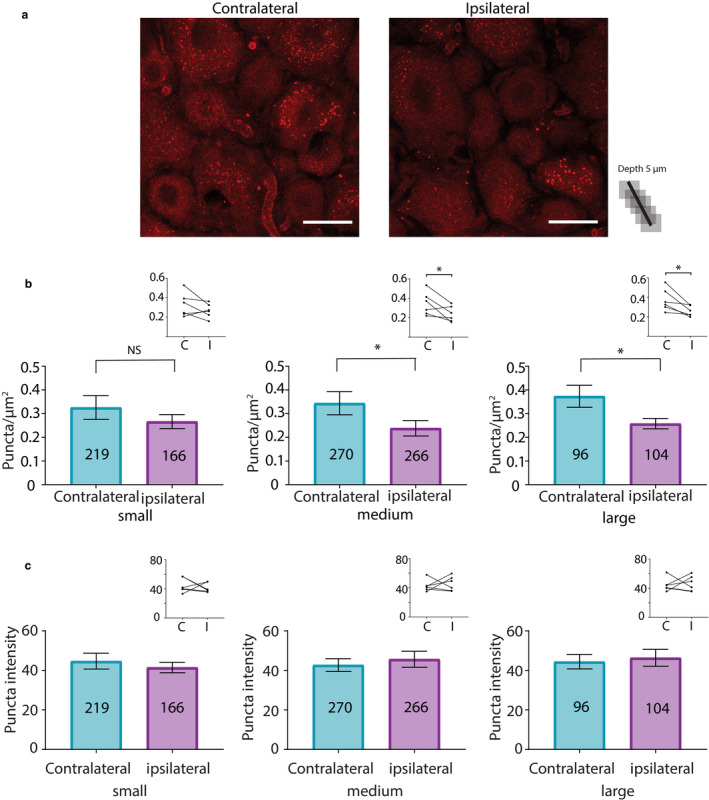
HCN2 channel SUMOylation by SUMO1 is diminished in medium and large diameter neurons from ipsilateral relative to contralateral DRG at 1 day post‐CFA. (a) Representative PLA. Scale bars are 25 µm. Note that this image provides an overview, but for the purpose of quantification, individual projections were made for each cell, for example, Figure 5b. (b) Plots of mean number of puncta/µm^2^ for all DRG from six CFA treated animals on day 1 post‐CFA The number inside the bar represents the total number of cells analysed. Inset: Each line compares means for contralateral and ipsilateral DRG from one animal. Paired *t*‐tests indicate medium and large diameter cells contain significantly fewer puncta in the ipsilateral DRG relative to the contralateral DRG (small, 0.33 ± 0.05 vs. 0.27 ± 0.03, *p* = 0.2058; medium, 0.34 ± 0.05 vs. 0.24 ± 0.03, *p* = 0.0480; large, 0.37 ± 0.05 vs. 0.26 ± 0.02, *p* = 0.0238), ^*^ *p* < 0.05. (c) Plots of mean puncta intensity on day 1 post‐CFA. Paired *t*‐tests indicate mean puncta intensities were not significantly different for any size category in ipsilateral compared to contralateral DRG (small, 44.68 ± 4.03 vs. 41.48 ± 2.66, *p* = 0.5986; medium, 42.7 ± 3.24 vs. 45.65 ± 4.06, *p *= 0.6299; large, 44.44 ± 3.68 vs. 46.39 ± 4.27, *p *= 0.7631). CFA, Complete Freund's Adjuvant; DRG, dorsal root ganglia; HCN2, hyperpolarization‐activated, cyclic nucleotide‐gated 2; PLA, proximity ligation assays; SUMO, small ubiquitin like modifier

Proximity ligation assays experiments were repeated for experimental and control animals except that anti‐SUMO1 was used. HCN2 channels decorated with SUMO1 were observed on day 1 post‐CFA (Figure 8). Paired *t*‐tests indicated that the mean number of HCN2‐SUMO1 puncta was not significantly different in small neurons from contralateral versus ipsilateral DRG, but puncta number was significantly reduced by ~30% in medium and large diameter neurons from ipsilateral relative to contralateral DRG (Figure 8b). There were no significant differences in mean puncta number between left versus right DRG from control animals for any cell size (small, 0.35 ± 0.06 vs. 0.34 ± 0.09, *p* = 0.7672; medium, 0.31 ± 0.05 vs. 0.27 ± 0.08, *p* = 0.3750; large, 0.39 ± 0.12 vs. 0.31 ± 0.07, *p* = 0.2184; *n* = 4). There were no significant differences in puncta intensity between contralateral versus ipsilateral DRG for any neuronal size (Figure 8c). There were no significant differences in puncta intensity for left versus right DRG from control animals (small, 55.22 ± 10.11 vs. 51.25 ± 7.35, *p* = 0.6250; medium, 55.83 ± 10.77 vs. 53.47 ± 6.94, *p* = 0.8750; large, 58.5 ± 11.15 vs. 55.65 ± 5.70, *p* > 0.9999; *n* = 4).

**Figure 9:**
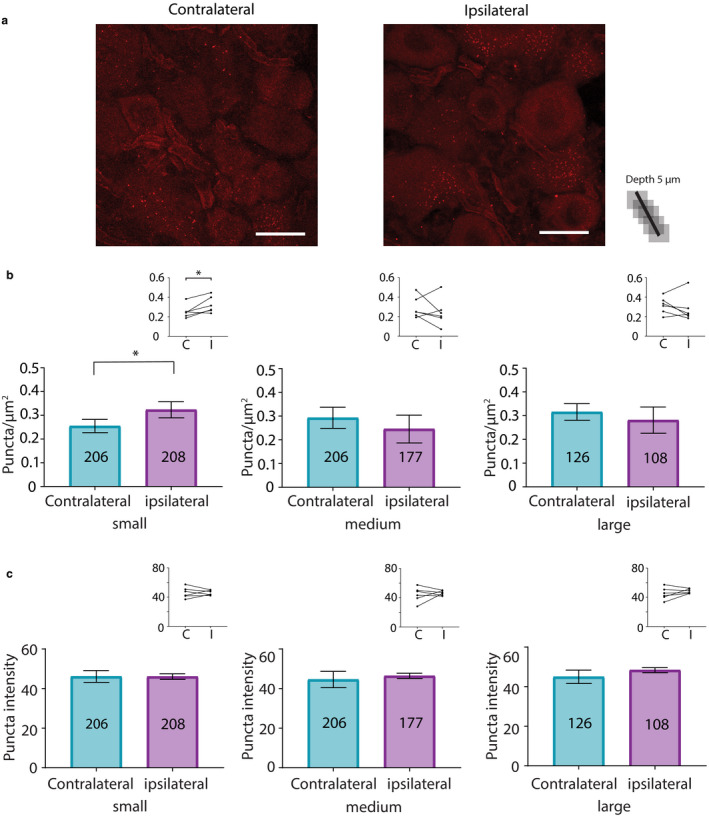
HCN2 channel SUMOylation by SUMO1 is increased in small neurons from ipsilateral relative to contralateral DRG at 3 days post‐CFA. (a) Representative PLA. Scale bars are 25 µm. Note that this image provides an overview, but for the purpose of quantification, individual projections were made for each cell, for example, Figure 4b. (b) Plots of mean puncta/µm^2^ for all DRG from six CFA treated animals on day 3 post‐CFA. The number inside the bar represents the total number of cells analysed. Inset: Each line compares means for contralateral and ipsilateral DRG from one animal. Significantly more puncta were observed in ipsilateral relative to contralateral DRG as indicated by paired *t*‐tests or non‐parametric alternative** (small, 0.25 ± 0.03 vs. 0.32 ± 0.03, *p* = 0.0285; medium, 0.29 ± 0.05 vs. 0.25 ± 0.06, *p* = 0.4310; large**, 0.32 ± 0.03 vs. 0.28 ± 0.06, *p* = 0.5625). *, p < 0.05 (c) Plots of mean puncta intensity on day 3 post‐CFA. Paired *t*‐tests indicate mean puncta intensities were not significantly different for any size category in ipsilateral compared to contralateral DRG (small, 46.14 ± 3.0 vs. 46.08 ± 1.41,* p* = 0.9826; medium, 44.58 ± 4.12 vs. 46.45 ± 1.32, *p* = 0.6612; large, 44.99 ± 3.38 vs. 48.35 ± 1.27 *p* = 0.2758). CFA, Complete Freund's Adjuvant; DRG, dorsal root ganglia; HCN2, hyperpolarization‐activated, cyclic nucleotide‐gated 2; PLA, proximity ligation assays; SUMO, small ubiquitin like modifier

SUMO1 conjugation to HCN2 channels was examined 3 days post‐CFA (Figure 9). HCN2‐SUMO1 puncta number was significantly increased by ~27% in small neurons from ipsilateral relative to contralateral DRG (Figure 9b). There were no significant differences in medium or large diameter neurons in ipsilateral relative to contralateral DRG (Figure 9b). There were no significant differences in puncta number for left versus right DRG from control animals (small, 0.28 ± 0.02 vs. 0.28 ± 0.08, *p* = 0.9861; medium, 0.23 ± 0.01 vs. 0.27 ± 0.10, *p* = 0.7612; large, 0.31 ± 0.03 vs. 0.33 ± 0.10, *p* = 0.8797; *n* = 3). As in all other cases, there were no significant differences in puncta intensity for contralateral versus ipsilateral DRG for any cell size (Figure 9c). Control animals also showed no differences in left versus right DRG puncta intensity (small, 65.55 ± 16.13 vs. 48.64 ± 4.701, *p* = 0.3539; medium, 71.14 ± 23.03 vs. 48.51 ± 3.7, *p* = 0.3559; large, 67.96 ± 18.46 vs. 50.43 ± 2.37, *p* = 0.3077; *n* = 3).

In sum, SUMO conjugation to HCN2 channels was increased in small diameter neurons from ipsilateral DRG on days 1 and 3 post‐CFA. Dynamic changes varied with the SUMO isoform. Whereas conjugation to HCN2 channels with SUMO2/3 increased on day 1, conjugation with SUMO 1 increased on day 3. In medium and large diameter neurons, SUMO1 conjugation to HCN2 channels was transiently reduced on day 1 post‐CFA.

## 4 DISCUSSION

An HCN2‐mediated increase in I_h_ is necessary for mechanical hyperalgesia experienced during persistent inflammation. The present study investigated how HCN2 channels in L5 DRG were altered during early stages of inflammation. The major finding of this study was that HCN2 protein expression was not sufficient for mechanical hyperalgesia. Moreover HCN2 channel SUMOylation was significantly increased in small diameter neurons and decreased in medium and large diameter neurons in ipsilateral relative to contralateral DRG.

### 4.1 HCN2 protein expression and sumoylation of HCN2 channel complexes are increased in small diameter neurons during CFA‐induced inflammation

Persistent inflammation is thought to be a primary mechanism whereby an acute injury leads to a chronic pain state (Dubin & Patapoutian, 2010; Emery, Young, & McNaughton, 2012; Pinho‐Ribeiro, Verri, & Chiu, 2017; Tsantoulas, Mooney, & McNaughton, 2016; Xu & Yaksh, 2011). CFA administration elicits persistent inflammation (Millan et al., 1988; Stein, Millan, & Herz, 1988). In rats, a unilateral, intraplantar CFA injection significantly reduces the mechanical force necessary to produce ipsilateral paw withdrawal 1–14 days after inflammation. The observed mechanical hyperalgesia is not present in the contralateral paw (Goff, Burkey, Goff, & Jasmin, 1998; Hurley & Hammond, 2000; Millan et al., 1988; Schnorr et al., 2014; Stein et al., 1988; Weng et al., 2012). The cutaneous inflammatory response is resolved by 17–21 days (Li et al., 2005). Inflammation alters several ionic currents in sensory neurons, including I_h_, and specifically increases the excitability of C‐fibre nociceptors (Herrmann et al., 2017; Pace et al., 2018; Takeda, Takahashi, & Matsumoto, 2014; Weng et al., 2012; Zemel, Ritter, Covarrubias, & Muqeem, 2018). I_h_ increases the excitability of DRG sensory neurons by limiting membrane hyperpolarization and facilitating depolarization (Yagi & Sumino, 1998). CFA‐induced inflammation triggers an increase in the activation kinetics and maximal conductance of nociceptor I_h_ (Djouhri et al., 2015). Injections of pharmacological agents to block I_h_ as early as 1 day after CFA‐induced inflammation prevent hyperalgesia (Schnorr et al., 2014; Weng et al., 2012).

The mechanism(s) producing a CFA‐induced increase in nociceptor I_h_ is unknown. In rat DRG sensory neurons, I_h_ is mediated largely by HCN isoforms 1 and 2, and to a lesser extent, HCN3 (Gao et al., 2012; Kouranova, Strassle, Ring, Bowlby, & Vasilyev, 2008; Weng et al., 2012), which can form homo‐ and hetero‐tetramers. Channel complexes can also contain auxiliary subunits (Piskorowski, Santoro, & Siegelbaum, 2011; Santoro et al., 2009, 2011; Saponaro et al., 2014). Genetic ablation of HCN2 from DRG sensory neurons attenuated mechanical hyperalgesia measured on day 3 post‐CFA (Schnorr et al., 2014). Hindlimb inflammation elicited a transient increase in HCN2 IR in small diameter neurons in ipsilateral rat L4 DRG on day 1 but not day 4 post‐CFA, relative to uninjected controls (Acosta et al., 2012). A significant decrease was also observed in large diameter neurons on day 4 post‐CFA (Acosta et al., 2012). A second study examined the effects of hindlimb inflammation on ipsilateral L5 DRG neurons 7 days post‐CFA; it showed a significant increase in HCN2 IR in small diameter neurons and a significant increase in the number of small and medium diameter neurons expressing HCN2, but no change in HCN1 or HCN3 expression relative to uninjected controls (Weng et al., 2012). The current study shows a significant bilateral increase in HCN2 staining intensity in small and medium diameter L5 DRG neurons on day 1 post‐CFA relative to uninjected controls. In addition, there is a significant bilateral increase in the number of cells expressing HCN2 on day 3 post‐CFA. We did not investigate if this increase in L5 DRG IR was accompanied by an increase in channel surface expression. Future studies will confirm if a CFA‐induced increase in nociceptor I_h_ is accompanied by an increase in nociceptor HCN2 surface expression. The bilateral changes were unexpected since CFA injection was unilateral. Unilateral peripheral nerve damage can result in unilateral hyperalgesia but bilateral changes in DRG cytokine and cytokine receptor mRNA and protein expression (Brazda, Klusakova, Svizenska, Veselkova, & Dubovy, 2009; Dubovy, Brazda, Klusakova, & Hradilova‐Svizenska, 2013; Jancalek, Dubovy, Svizenska, & Klusakova, 2010; Jancalek, Svizenska, Klusakova, & Dubovy, 2011) and NaV1.8 mRNA (Oaklander & Belzberg, 1997). Such changes are thought to be due to a generalized inflammatory response. Several mechanisms could underlie bilateral effects (Koltzenburg, Wall, & McMahon, 1999).

Our data suggest that an increase in HCN2 protein expression is not sufficient for mechanical hyperalgesia because, unilateral injection of CFA produces unilateral hyperalgesia (Hurley & Hammond, 2000; Stein et al., 1988) but a bilateral increase in HCN2 protein expression (Figures 2 and 3). Altered expression of additional ion channels and/or other proteins may be necessary for hyperalgesia (Pace et al., 2018). Alternatively, additional post‐translational modifications to HCN2 channels may be required. Recent studies demonstrated that enhanced ion channel SUMOylation can contribute to hyperalgesia in models of chronic pain (Francois‐Moutal et al., 2018; Moutal et al., 2017; Wang et al., 2018a). Post‐translational SUMOylation can regulate ion channel biophysical properties, trafficking and surface expression (Benson et al., 2007; Chamberlain et al., 2012; Dai, Kolic, Marchi, Sipione, & Macdonald, 2009; Dustrude et al., 2016; Martin, Nishimune, Mellor, & Henley, 2007; Parker et al., 2017; Plant, Zuniga, Araki, Marks, & Goldstein, 2012; Plant et al., 2010, 2016; Qi et al., 2014; Rajan et al., 2005; Steffensen et al., 2018; Wang et al., 2018b; Welch et al., 2019; Xiong et al., 2017). Our data indicate that unilateral CFA injection produces unilateral hyper‐SUMOylation of HCN2 in small diameter neurons from L5 DRG. HCN2 SUMOylation produces an increase in channel surface expression and I_h_ in a heterologous expression system (Parker et al., 2017), but the effect of enhanced DRG HCN2 SUMOylation observed here is unknown.

Because HCN2 α‐subunits are SUMOylated in the DRG (Figure 4), the most parsimonious interpretation of the PLA experiments is that SUMOylation of HCN2 α‐subunits is enhanced during inflammation. However, in order to produce a fluorescent punctum in a PLA, the HCN2 and SUMO proteins need to be within 40 nM of one another so that their antibodies can interact to generate a punctate fluorescent signal. HCN channels can exist as heterotetramers in multiprotein complexes with auxiliary proteins like TRIP8b (Santoro et al., 2009) and MINT (Kimura, Kitano, Nakajima, & Nakanishi, 2004), which also have predicted SUMOylation sites. This means that the increase in PLA puncta number could also represent increased SUMOylation of other HCN isoforms and/or auxiliary subunits that make‐up the HCN2 channel complex. Indeed, it is well documented that a cell stressor can evoke group SUMOylation, whereby several proteins in a physically linked group are non‐specifically SUMOylated, as opposed to targeting one specific protein within the group (Jentsch & Psakhye, 2013).

The increase in SUMOylated HCN2 channel complexes is a modest ~27%, but it is fairly common for low levels of SUMOylation to exert substantial regulatory effects (Benson et al., 2007; Flotho & Melchior, 2013). Nevertheless, there are examples of much greater increases in SUMOylation, for example, CRMP2 SUMOylation in the sciatic nerve, spinal dorsal horn and glabrous skin appears to increase by as much as ~300% in a rat model of neuropathic pain (Moutal et al., 2017). Small diameter DRG neurons can be classified into at least six cell types (Li et al., 2016; Usoskin et al., 2015), and it is possible that a specific nociceptor cell type representing a small fraction of the population shows a much larger increase in HCN2 SUMOylation. Since the cell type(s) exhibiting the CFA‐induced enhancement of HCN2 protein expression and HCN2 channel SUMOylation were not identified, we cannot conclude that increased expression and SUMOylation are coincident in the same cell or that the inflammation‐induced increase in nociceptor I_h_ depends upon a two‐step process.

There are four SUMO isoforms and three were examined in this study. Conjugation of SUMO2/3 versus SUMO1 to HCN2 channel complexes was differentially regulated as inflammation progressed: SUMO2/3 but not SUMO1 conjugation to HCN2 channels was significantly increased on day 1 post‐CFA, and the opposite was true on day 3 post‐CFA. The sole SUMO conjugating enzyme, ubc9, does not show a preference for SUMO isoforms and interacts with a similar affinity for all isoforms (Knipscheer, van Dijk, Olsen, Mann, & Sixma, 2007), but the SENP family of SUMO isopeptidases that mediate SUMO deconjugation can exhibit SUMO isoform specificity and can be differentially localized by post‐translational modification (Kunz et al., 2018). Likewise, diverse E3 proteins that stabilize interactions between ubc9 and its targets can also exhibit SUMO isoform specificity (Cappadocia, Pichler, & Lima, 2015; Koidl et al., 2016; Tatham et al., 2005). Together, these data support the emerging ideas that inflammatory mediators regulate the nociceptor SUMOylation machinery and that this regulation may effect alterations in multiple ionic conductances during nociceptor sensitization.

### 4.2 Sumoylation of HCN2 channel complexes is decreased in medium and large diameter neurons during CFA‐induced inflammation

SUMO1 conjugation to HCN2 channel complexes was transiently decreased in medium and large diameter neurons from ipsilateral L5 DRG on day 1 of inflammation. This regulation could potentially contribute to hyperalgesia which peaks on day 1 (Millan et al., 1988; Stein et al., 1988). Medium and large neurons could contribute directly to hyperalgesia as nociceptors (Dubin & Patapoutian, 2010; Nagi et al., 2019), or they could indirectly influence nociceptor activity through presynaptic mechanisms and/or other circuit effects (Braz, Solorzano, Wang, & Basbaum, 2014; Guo & Hu, 2014; Mendell, 2014). It will be important to identify these neurons and how HCN2 channel deSUMOylation regulates their activity.

## CONFLICT OF INTEREST

None declared.
